# Impact of anti-cancer drugs and other determinants on serum protein binding of morphine 6-glucuronide

**Published:** 2010

**Authors:** S.O. Mashayekhi, M. Ghandforoush-Sattari, D.C. Buss, P.A. Routledge, R.DW. Hain

**Affiliations:** 1 National public Health Management research center (NPMC), Department of Clinical Pharmacy; 2 Haematology & Oncology Research Centre, Department of Pharmacology and Toxicology, Faculty of Pharmacy, Tabriz University of Medical Sciences, Tabriz, Iran; 3 Department of Pharmacology, Therapeutics and Toxicology, Cardiff University; 4 Department of Child Health, Llandough Hospital, College of Medicine, UK

**Keywords:** Albumin, Morphine, Morphine 6-glucuronide, Protein binding

## Abstract

**Background and the purpose of the study:**

The aim of the present study was to examine factors that may influence the protein binding of morphine 6-glucuronide (M6G), the most active metabolite of morphine.

**Methods:**

An enzyme-linked immunoabsorbent assay technique was used to measure the M6G concentration in serum of 18 healthy adults, 18 neonatal and 7 children with cancer. Total and free M6G concentrations were measured following equilibrium dialysis for 3 hrs and at physiological pH at 37°C. The influence of vincristine, methotrexate, 6-mercaptopurine, morphine, human albumin, alpha-1-acid glycoprotein, palmitic acid, oleic acid and pH on M6G protein binding was examined.

**Results:**

M6G was 66.87±0.73 percent free in human serum at physiological pH and temperature. The percentage free (unbound) was increased significantly by vincristine (4.33%) and methotrexate (9.68%), but 6- mercaptopurine and morphine had no significant effect on it. Free percentages of M6G was reduced by decreasing serum albumin concentration but was unaffected by the presence of alpa-1-acid glycoprotein (AAG) or changes in serum pH. Similar results were obtained in human serum albumin (HAS) solutions. Addition of palmitic acid and oleic acid reduced protein binding significantly by 6.3% and 7.4%, respectively.

**Major conclusion:**

Although M6G in this study was not highly bounded, but because of its high analgesic potency, any change in its free concentration due to concurrent medication or disease caused significant changes in its effects. This dearth of evidence has been implicated in the reluctance of professionals to be cautious in prescribing them to children, particularly in the neonatal period.

## INTRODUCTION

Morphine is conjugated in the liver to morphine-6- glucuronide (M6G, 10–15%) and morphine-3- glucuronide (M3G, 45–55%) (1). The analgesic potency of M6G is significantly greater than that of morphine by itself (2). It has been suggested that it is the major contributor to analgesia following morphine administration (3). Morphine is often co-prescribed with a number of drugs including those with high protein binding and as a result in patients who receive morphine M6G protein binding could be influenced.

Previous studies have shown that M6G pharmacokinetics in children differ from adults (4) and considering the potential role of M6G in clinical situations, it is surprising that little is known about its potential interactions with other drugs. Previously measurement of M6G protein binding was performed in a pooled plasma (5) and five plasma samples (6) without studying any determinants. Very little is known on the extent to which M6G is bound to protein in plasma and its binding determinants. This is potentially important when morphine is co-prescribed with anti- cancer drugs. The aim of this study was to characterise the protein binding of M6G in children and neonates in health and cancer states, and to assess the impact of protein binding of some common antineoplastic agents and other determinants on M6G protein binding.

## MATERIALS AND METHODS

### Materials

Morphine 6-glucuronide (Sigma-Aldrich, UK), human serum albumin (HSA, Sigma-Aldrich, UK), human alpha -acid glycoprotein (AAG, Sigma- Aldrich, UK), palmitic acid (Sigma Chemical Company), oleic acid (BDH Chemicals Ltd), Teflon equilibrium dialysis cells (MSD Dianorm), and Spectrapor® 2 membrane (molecular weight cut- off, 12-14,000 daltons) were used in this study. Sorensen's phosphate buffer of 0.15 M containing 0.59% sodium chloride with pH of 7.4 was prepared by the described method (7).

### Methods

M6G concentration was measured by enzyme-linked immunosorbent assay (ELISA) (8). Serum free percentage of M6G was determined by equilibrium dialysis similar to determination of morphine protein binding (9). The examined concentration of M6G was within the therapeutic concentration range after morphine administration (4). One ml aliquots of spiked Sorensen's buffer were added to Teflon equilibrium dialysis cells and dialyzed for 3 hrs at 37°C against one ml aliquots of serum or protein solution. The two compartments were separated by a Spectrapor® 2 membrane. Any leakage or adhesion of the drug during the experiments was identified by measurment of albumin concentration in the protein compartment before and after dialysis and determination of M6G concentration in the chambers of the dialysis apparatus before and after dialysis and comparison of their total concentration with the originally added M6G. Equilibrium was achieved within 120 min and remained stable for 180 min. Samples were run in duplicates. The percentages of unbound drug were calculated as percentages of the ratio of concentrations in the solutions of buffer and serum at the end of the dialysis.

Serum albumin concentrations were measured using Albumin Reagent (BCP) purchased from Sigma Diagnostics®. AAG concentrations were measured using “NOR Partigen® α1 -acid glycoprotein(Dade Behring) immunodiffusion plates

#### Determination of M6G protein binding in healthy adult subjects, neonates and children on treatment for cancer

Eighteen healthy, drug free, non-fasting subjects were enrolled in the study. Blood was collected by direct venepuncture into polypropylene syringes into anticoagulant-free glass tubes. The samples were centrifuged 30 min after collection at 3000 rpm for 15 min. The serum albumin and AAG concentrations were measured immediately. The serum samples were frozen at -20°C in glass tubes. Samples were thawed within 1 week for estimation of protein binding. PH of the serum samples were measured immediately before and after dialysis

Neonatal blood sample collection was carried out at the Delivery Suite in Llandough Hospital. Blood was collected form the umbilical cord of newborn babies. Mothers who had received any opioid before or during delivery were excluded from the study. Samples were prepared by the same procedure which was explained before.

Blood samples collected from children with cancer who received morphine as part of their treatment were used in the present study. After collection of samples and separation of the serum, a serum sample was applied to the normal procedure of protein binding measurement. The demographic information for children with cancer has been reported previously (4).

The research was conducted in accordance with the Declaration of Helsinki and the required permission for collection of blood from healthy adults, the neonatal and children was received from Bro Taf Health Authority in Cardiff.

#### Determination of effects of human serum albumin, AAG and nonesterified fatty acids (NEFAs) on M6G binding

In vitro studies were performed using HSA made up in a solution of Sorensen's buffer (2.5, 5, 10, 20, 40, 60, and 70 g/l) (9). The buffer was spiked with 50 ng/ml of M6G.

Pure AAG (1.0 and 2.0 g/l), oleic acid (1.0 mmol) and palmitic acid (1.0 mmol) were added to a solution of 40 g/l of HSA separately. Six cells of each solution were prepared and dialyzed against spiked Sorensen's phosphate buffers with 50 ng/ml of M6G. Control solutions were treated in an identical manner (9).

#### Determination of effect of pH and M6G concentration on M6G binding

The effects of pH on serum protein binding of M6G were measured in the serum of a healthy volunteer. Serum samples were adjusted by HCl or NaOH to the desired pH values (7.0, 7.2, 7.4, 7.6, 7.8 and 8.0) and dialyzed against spiked Sorensen's phosphate buffers with 50 ng/ml of M6G of the same pH values. Other serum aliquots were left untreated and dialyzed against a range of phosphate buffer of pH between 7.0 and 8.0 (7.0, 7.2, 7.4, 7.6, 7.8 and 8.0). M6G was added to the buffer to achieve pre-dialysis concentrations between 5 and 200 ng/ml. Equilibrium dialysis was performed against serum of a healthy volunteer. M6G concentrations were measured in the serum chambers and their correlation with free M6G percentages was examined (9).

#### Determination of disposition of M6G by anti cancer drugs and morphine

The effects of three anti-cancer drugs; vincristine, 6- mercaptopurine (6MP) and methotrexate on protein binding of M6G, were studied and compared with a control sample of M6G (0 µg/ml of anti cancer drugs). The reported maximum concentration of vincristine, 6MP and methotrexate in the plasma of adults receiving these agents were 2260±212 ng/ ml (10), 653±344 ng/ml (11) and 5 µM (12), respectively. The protein binding of these agents are reported to be 71% (13), 20- 90% (14) and 25-55% (15), respectively. In order to study the effects of therapeutics and toxic concentration of these drugs, three concentrations of each drug, one within normal plasma concentration, one close to the highest plasma concentration and one higher than maximum plasma concentration were chosen. Possible effect of morphine which is also present in the plasmaon M6G protein banding was also examined (9). None of the examined anti-cancer medications were administered to the children with cancer at the time of blood collections.

#### Comparison of protein binding of M6G in serum and plasma sample

Ten ml blood from one volunteer was collected into a lithium heparinised tube and another 10 ml added to a plain glass tube with no anti-coagulant. Both samples were centrifuged at 3000 rpm for 15 min before storage at -20° C. Equilibrium dialysis was performed in six cells for each sample (9).

#### Determination of the Effect of storage of blood and buffer samples on M6G binding

In order to investigate the effect of storage of serum samples at -20°C, blood from a non-fasting, drug free, healthy volunteer was collected. The serum was divided into 5 parts. Buffer was spiked with 50 ng/ml of M6G which also divided into 5 parts. The first serum sample and buffer were applied to the equilibrium dialysis on the day of collection. The other samples were kept at -20° C until they were dialyzed after 7, 14, 21 and 28 days.

#### Statistical analyses

Statistical calculations were carried out using the computer software package “Prism”. ANOVA with Dunnett's multiple comparison tests and unpaired t test analysis were used to detect significant correlation between variables. Probability values of P<0.05 were considered to be significant.

## RESULTS

### 

#### M6G protein binding in healthy adult subjects, neonates and children following treatment with anti- cancer drugs

The percentage of free M6G in the serum of the 18 adult subjects (10 male and 8 female) aged from 24 to 53 years was 61.72%±2.94. Their serum albumin concentration ranged from 33.0 to 51.0 g/l (43.8±4.4 g/l). The serum AAG was 0.8±0.3 g/l and varied from 0.333 to 1.42. Albumin was the main binding protein for M6G. The binding ratio (*bound/free*) of M6G in these subjects was significantly related to albumin concentrations (R2=0.506, P=0.0009). The relationship between free percentages of M6G and AAG concentration in the subjects failed to reach statistical significance (R2=0.198, P=0.0644). There was no significant relationship between free percentages of M6G and serum pH before and after dialysis (R2=0.001, P=0.8932 and R2=0.087, respectively, P=0.2340). No significant correlation was found between subjects’ age and M6G free percentages (R2=0.180, P=0.0793). Multiple regression analysis of effects of serum albumin and AAG concentration on M6G binding showed that there was a significant relationship between their concentrations and M6G free percentages in adult subjects (R2=0.543, P<0.05) and effects of serum albumin concentration on M6G protein binding was significantly greater than effects of AAG on M6G protein binding.

In neonatal samples, serum albumin and AAG concentration were 32.4±3.0 g/l, 0.32±0.1 g/l, respectively. M6G protein free percentages in neonatal samples was 70.5%±3.8 and changed significantly by changes in serum albumin concentration (R2=0.407, P=0.0044). There was also a significant relationship between serum albumin concentration and the binding ratio of M6G (R2=0.420, P=0.0036). Unlike albumin, there was no significant relationship between M6G free percentages and serum AAG concentration (R2=0.069, P=0.294) or serum pH, either before or after dialysis (R2=0.200, P=0.0626 and R2=0.250, P=0.0544, respectively). Multiple regression analysis of effects of serum albumin and AAG concentration on M6G binding percentages showed that there was a weak but significant relationship between their concentrations and M6G free percentages in neonates subjects (R2=0.408, P=0.02) and effects of serum albumin concentration on M6G protein binding were significantly greater than that of AAG concentration in serum on binding.

The free percentage of M6G in serum of children with cancer was 69.20±4.26 and ranged from 62.33 to 77.72%. It was significantly correlated to serum albumin concentration (R2=0.607, P=0.039) but there was no significant relationship with serum AAG concentration (R2=0.092, P=0.509) or serum pH before and after dialysis (R2=0.009, P=0.8360 and R2=0.202, P=0.2018, respectively). The M6G binding ratio was significantly related to serum albumin concentration in children (R2=0.644, P=0.03). Furthermore, M6G free percentages was not significantly related to its concentration after dialysis (R2=0.019, P=0.769).

Multiple regression analysis of effects of serum albumin and AAG concentration on M6G free percentages showed that there was a significant relationship between these parameters in children patients (R2=0.408, P=0.02) and the effect of serum albumin concentration on M6G protein binding was significantly greater than AAG serum concentration in serum on binding.

#### Effects of Human Serum albumin, AAG and nonesterified fatty acids (NEFAs) on M6G binding

**Figure 1:**
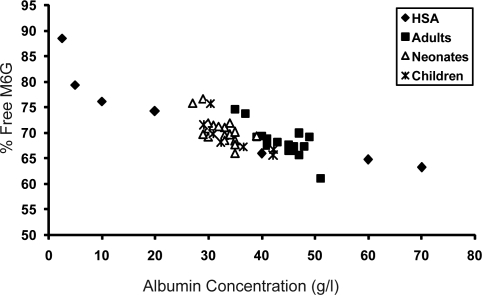
The relationship between the percentage of free M6G to protein and the concentration of human serum albumin solution and in healthy volunteers, neonates and children serum.

**Figure 2:**
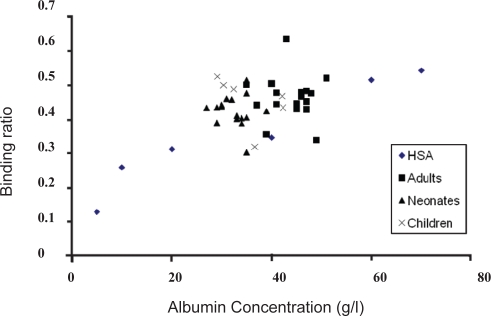
The relationship between the ratio of bound to free (B/F) for M6G and the concentration of human serum albumin solution in serum of healthy subjects and human serum albumin solutions of varied concentrations.

A study of the effect of different concentrations of HSA in Sorensen's buffer on M6G binding showed that increasing HSA concentration decreased M6G free percentage (R2=0.827, P=0.0082, Figure 1) so that thee binding ratio of bound to free drug (B/F) of M6G in human serum albumin solutions was related to albumin concentration up to 70 g/l (R2=0.891, P=0.001, Figure 2). The calculated percentage of free M6G at 43.8 g/l of HSA concentration (the mean albumin concentration in the 18 subjects) was 68.8%, which was very similar to the free percentage in the healthy subjects with equal serum albumin concentration (67.7%).

Addition of AAG (1.0 or 2.0 g/l) did not alter the percentages of M6G in the free form (64.0%±1.5 and 64.1%±1.6 and 67.5%±1.6; P<0.01, respectively). Addition of palmitic acid (1.0 mmol/l) to HSA (40 g/l) increased the free percentage of M6G from 67.5%±1.6 to 73.8%±2.2 (P<0.01) and the same concentration of oleic acid produced a slightly larger increase from 67.5%±1.6 to 74.9%±0.6 (P<0.01).

#### Effect of pH and M6G concentration on M6G binding

Free M6G was unaffected by pH in either the untreated serum (R2=0.340, P>0.05) or the serum, which had been adjusted with acid or alkali prior to dialysis (R2=0.261, P>0.05). These points when combined and subjected to unpaired t test analysis revealed no significant difference between the regression lines (P>0.05).

**Figure 3:**
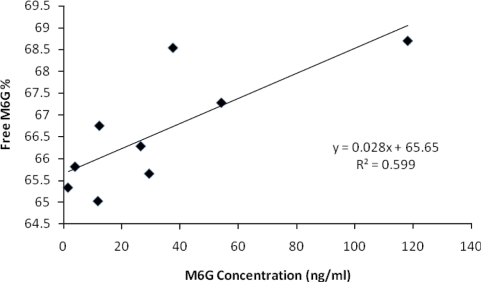
The effect of M6G concentration on free M6G.

In serum from one volunteer the mean percentage of free drug between 1.63 and 118.1 ng/ml was 66.4%±1.7 (n=9). The relationship between percentage of free M6G and total serum M6G concentration was significant (R2=0.600, P=0.014). Serum protein binding of M6G was linearly but negatively related to M6G concentration over this range of concentrations (Figure 3).

#### Disposition of M6G by anti-cancer drugs and morphine

**Figure 4:**
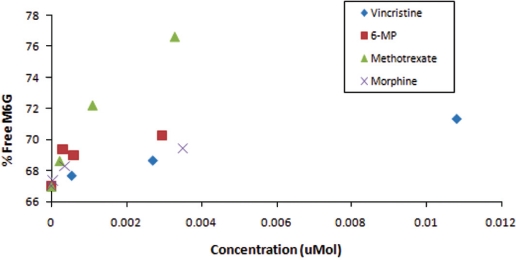
The effect of anti-cancer drugs on free M6G.

Increase in the concentration of vincristine caused an increase in the free M6G from 67.0%±0.9 to 71.33%±1.6 (R2=0.978, P=0.0113). Increasing the methotrexate concentration up to 1.5 µg/ml raised free percentage of M6G to 76.7%±3.2 (R2=0.952, P=0.0243). Further increase in the concentration of these drugs, might result a larger raise in free M6G percentages. For morphine and 6-MP, no significant effect was observed, which means a larger increase in their concentrations are unlikely result in further alteration in protein binding of M6G (R2=0.831, P=0.0883, and R2=0.557, P=0.2535, respectively). These results are shown in figure 4.

#### Protein binding of M6G in serum and plasma samples

There was no significant difference between the serum and plasma protein binding of M6G (66.7%±1.4 vs. 67.95%±3.0 respectively, P>0.05).

#### Effect of storage of blood and buffer samples on M6G binding

Storage of serum and spiked buffer at -20°C for 7, 14, 21 and 28 days before equilibrium dialysis did not have any significant effect on serum bound M6G when compared with the same day collection and dialysis of the samples (P>0.05).

## DISCUSSION

Only the unbound fraction of drug exerts its clinical effects. Potentially, the protein binding of M6G could have an impact on its clinical use. Despite this, little is known about protein binding of M6G in adulthood, infancy or childhood. Other drugs such as tubocurarine, metocurine, propranolol, and lidocaine (16) have shown to have lower protein binding in neonatal blood than adult. Neonates may be at particular risk from changes in free drug concentration because of immaturity of the heart and the blood brain barrier (17). In this study it was attempted to simulate conditions of in vitro more closely and to assess some of the factors with potential impact on the clinical effects of M6G. These factors were selected cancer chemotherapy agents, concentrations of the binding proteins; albumin and AAG as well as nonesterified fatty acids (NEFA).

The percentage of free M6G in serum of healthy adults was slightly lower than results of a study of M6G protein binding in a smaller number of subjects (63.3%±3.8, n=8) (5) but significantly lower than the result of another study which reported 89%±2 free M6G percentages in pooled plasma from 5 volunteers (6). Since neither of studies provided information on their subjects’ protein levels or drug concentration, it is difficult to compare directly the results of the reported studies with those of this study. However, Vree and co-workers study was performed at room temperature rather than at 37 °C and this tends to result in an increase in the protein binding of many drugs as previously reported in cases of morphine (5) and phenytoin (18). While lower level of binding reported by Milne and co- workers could be due to binding to the dialysate apparatus, there is no information on this matter. The difference could be because of the used methods. Unlike the present study, in both studies, an Amicon micropartition ultracentrifuge system MPS-1 was used for measurement of plasma protein binding of M6G. In the present study, there was no binding to the membrane or the equilibrium dialysis cells and also there also was not any leakage of protein or buffer solutions to the other compartments or to the water bath.

Previous study on morphine protein binding (9) showed that its protein binding in adults, neonates and children with cancer was significantly lower than M6G free percentages of the present study. M6G is a more polar compound than morphine itself and as it has been found for other similar compounds, the free percentage was higer (5).

Albumin appears to be the major binding protein of M6G and AAG has a much weaker role in M6G protein binding. One of the important biological activities of AAG is the ability to bind numerous basic and neutral lipophilic drugs such as propranolol (19) and tricyclic antidepressants (20). In vitro studies provided evidence of two classes of binding sites for basic and neutral drugs on AAG (21). The extent of binding of drugs to AAG depends on several factors such as the conformational changes of AAG, polarity of ligands, temperature, or pH (22). The lower binding of M6G to AAG could be of any of the above factors.

The degree of binding in HSA was very similar to that of serum at an equivalent albumin concentration. The low correlation between M6G free percentages and albumin concentration in the healthy volunteers than in the study using HSA alone may be related to the narrower range of albumin concentrations observed in the subjects, possibly together with effects of other factor(s) such as NEFA concentrations. The relationship between the ratio of bound to free (binding ratio) for M6G and the concentration of human serum albumin solution was significantly related to samples of HSA solution, healthy adults, neonates and children, implying a constant number of binding sites for M6G on HSA molecules and that the dissociation constant of the drug protein complex is much greater than the free drug concentration (23). In contrast to expectation, addition of AAG even in concentrations higher than that reported in male aged 20-25 years old (0.5-1.17 g/l (7)) did not alter the binding in the expected direction. This is in marked contrast to that of many weakly basic drugs such as fentanyl and alfentanil (24), which bind primarily to AAG.

Total NEFA in females and male aged 20-25 years old were reported to be 293±151 and 251±114 µM, respectively (7). Palmitic acid and oleic acid each make up about one third of the total serum NEFA concentration. NEFA have been reported to associate with albumin (25), which generally contribute to increase of the serum free percentages of many drugs in serum (26). In this study it was shown as a small but significant raise in free percentages of M6G in the presence of palmitic acid and oleic acid (P<0.01). NEFA may thus be a determinant of M6G binding, although the magnitude of their effects on health is likely to be small.

It was hypothesized that M6G serum free percentages could be higher increase in drug concentration because of the higher ratio of drug concentration to proteins concentration in serum. It was only possible to confirm a small but statistically significant raise in free M6G by increasing its concentration. It is possible that higher concentrations might have some significant effects, but such concentrations are unlikely to be achieved clinically. There was a 3.4% change in free percentages of M6G when there was an increase in serum concentration from 1.56 to 118.10 ng/ml. For every one ng/ml increase in serum concentration of M6G there was a 0.03% increase in M6G free percentages. It seems that changes in concentration alter more morphine protein binding than that of M6G (about 5 times more) (9). The effect of concentration protein binding of morphine and M6G together could have additional effects on analgesic properties of morphine.

A comparison of M6G protein binding in serum and plasma showed that presence or absence of fibrinogen in the sample did not alter binding in any way, suggesting lack of binding to fibrinogen. In vitro addition of heparin to plasma did not affect the protein binding of M6G, which was similar to that reported by Spector (26) for some other drugs. Morphine and 6-MP concentrations did not cause a significant change in binding of M6G. These results suggest that either morphine and M6G have different binding sites, or there are enough protein or unoccupied binding sites for morphine and M6G and as a result they do not displace each other. On the other hand, 6-MP could not replace M6G which mean that the two compounds have different binding sites, bind to different serum proteins, or the presence of enough protein or unoccupied binding sites for M6G and 6-MP. Increasing concentrations of vincristine and methotrexate increased M6G free percentages significantly, indicating that these anti-cancer drugs may bind to the same binding site on the same protein or cause conformational changes at those binding sites. It is unlikely that these drugs could have a clinically important effect on M6G pharmacokinetics or phramacodynamics. It might be possible that M6G has a clinically significant displacement effect on these anti-cancer medications which was not investigated. The latter is especially true when M6G effects are added to the effects of other cancer medications such as vinblastine, cyclophosphamide, 5-fluorouracil, prednisone. These are sometimes co-prescribed with vincristine or/and methotrexate. It is also possible that there may be a greater effect when high doses of vincristine and methotrexate is used in cancer treatment.

## References

[1] Yeh SY, Gorodetzky CW, Krebs HA (1977). Isolation and identification of morphine 3- and 6-glucuronides, morphine 3,6-diglucuronide, morphine 3-ethereal sulfate, normorphine, and normorphine 6-glucuronide as morphine metabolites in humans. J Pharm Sci.

[2] Osborne R, Thompson P, Joel S, Trew D, Patel N, Slevin M (1992). The analgesic activity of morphine-6- glucuronide. Br J Clin Pharmacol.

[3] Osborne R, Joel S, Trew D, Slevin M (1988). Analgesic activity of morphine-6-glucuronide. Lancet.

[4] Mashayekhi SO, Ghandforoush-Sattari M, Routledge PA, Hain RD (2009). Pharmacokinetic and pharmacodynamic study of morphine and morphine 6-glucuronide after oral and intravenous administration of morphine in children with cancer. Biopharm Drug Dispos.

[5] Vree TB, Verwey-van Wissen CP (1992). Pharmacokinetics and metabolism of codeine in humans. Biopharm Dispos.

[6] Milne RW, Nation RL, Somogyi AA, Bochner F, Griggs WM (1992). The influence of renal function on the renal clearance of morphine and its glucuronide metabolites in intensive-care patients. Br J Clin Pharmacol.

[7] (1981). Geigy Scientific Tables.

[8] Mashayekhi S, Ghandforoush-sattari M, Hain R, Nixon L (2007). Morphine-6-glucuronide assay using a competitive ELISA assay. Pharmaceutical Sciences.

[9] Mashayekhi SO, Hain RDW, Buss DC, Routledge PA (2007). Morphine in children with cancer: impact of age, chemotherapy and other factors on protein binding. Journal of Pain & Palliative Care Pharmacotherapy.

[10] Embree L, Gelmon K, Tolcher A, Hudon N, Heggie J, Dedhar C, Logan P, Bally MB, Mayer LD (1998). Pharmacokinetic behavior of vincristine sulfate following administration of vincristine sulfate liposome injection. Cancer Chemother Pharmacol.

[11] Kato Y, Matsushita T, Chiba K, Hijiya N, Yokoyama T, Ishizaki T (1991). Dose-dependent kinetics of orally administered 6-mercaptopurine in children with leukemia. J Pediatr.

[12] Lawrenz-Wolf B, Wolfrom C, Frickel C, Fengler R, Wehinger H, Henze G (1994). [Severe renal impairment of methotrexate elimination after high dose therapy]. Klin Padiatr.

[13] de Lannoy IA, Mandin RS, Silverman M (1994). Renal secretion of vinblastine, vincristine and colchicine in vivo. J Pharmacol Exp Ther.

[14] Sjoholm I, Stjerna B (1981). Binding of drugs to human serum albumin XVII: Irreversible binding of mercaptopurine to human serum proteins. J Pharm Sci.

[15] Combe B, Edno L, Lafforgue P, Bologna C, Bernard JC, Acquaviva P, Sany J, Bressolle F (1995). Total and free methotrexate pharmacokinetics, with and without piroxicam, in rheumatoid arthritis patients. Br J Rheumatol.

[16] Wood M, Wood AJ (1981). Changes in plasma drug binding and alpha 1-acid glycoprotein in mother and newborn infant. Clin Pharmacol Ther.

[17] Lerman J, Strong HA, LeDez KM, Swartz J, Rieder MJ, Burrows FA (1989). Effects of age on the serum concentration of alpha 1-acid glycoprotein and the binding of lidocaine in pediatric patients. Clin Pharmacol Ther.

[18] Kodama H, Kodama Y, Itokazu N, Shinozawa S, Kanemaru R, Sugimoto T (2001). Effect of temperature on serum protein binding characteristics of phenytoin in monotherapy paediatric patients with epilepsy. J Clin Pharm Ther.

[19] Tiula E, Tallgren LG, Neuvonen PJ (1987). Serum protein binding of phenytoin, diazepam and propranolol in chronic renal diseases. Int J Clin Pharmacol Ther Toxicol.

[20] Ma Y, Henry JA (2001). The antidotal effect of alpha(1)-acid glycoprotein on amitriptyline toxicity in cardiac myocytes. Toxicology.

[21] Schley J, Muller-Oerlinghausen B (1986). Investigation of the binding of various tricyclic neuroleptics and antidepressants to alpha 1-acid glycoprotein. J Pharm Pharmacol.

[22] Fournier T, Medjoubi NN, Porquet D (2000). Alpha-1-acid glycoprotein. Biochim Biophys Acta.

[23] Nilsen OG, Leren P, Aakesson I, Jacobsen S (1978). Binding of quinidine in sera with different levels of triglycerides, cholesterol, and orosomucoid protein. Biochem Pharmacol.

[24] Wilson AS, Stiller RL, Davis PJ, Fedel G, Chakravorti S, Israel BA, McGowan FX (1997). Fentanyl and alfentanil plasma protein binding in preterm and term neonates. Anesth Analg.

[25] Coyle DE, Denson DD, Essell SK, Santos DJ (1986). The effect of nonesterified fatty acids and progesterone on bupivacaine protein binding. Clin Pharmacol Ther.

[26] Spector AA, Santos EC, Ashbrook JD, Fletcher JE (1973). Influence of free fatty acid concentration on drug binding to plasma albumin. Ann N Y Acad Sci.

